# Supplementary figures and images for: Efficient and controlled domain wall nucleation for magnetic shift registers
Source: Sci Rep. 2017 Sep 19;7:11909. doi: 10.1038/s41598-017-12230-9 (PMC5605553; doi:10.1038/s41598-017-12230-9)

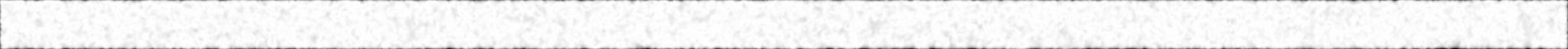

Supplement: Supplementary file 1 — Video Fig. 5a [file 41598_2017_12230_MOESM1_ESM.gif]

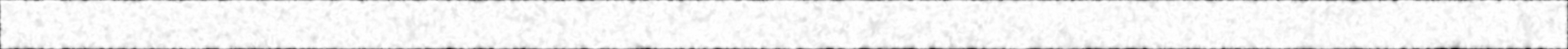

Supplement: Supplementary file 2 — Video Fig. 5b [file 41598_2017_12230_MOESM2_ESM.gif]
