# Supplementary material for: Efficient and controlled domain wall nucleation for magnetic shift registers
Source: Sci Rep. 2017 Sep 19;7:11909. doi: 10.1038/s41598-017-12230-9 (PMC5605553; doi:10.1038/s41598-017-12230-9)
Supplement: Supplementary file 3 — Supplementary Information [file 41598_2017_12230_MOESM3_ESM.pdf]

Supplementary information: Efficient and controlled domain wall nucleation for magnetic shift-registers

Oscar Alejos[1], Víctor Raposo[2], Luis Sánchez-Tejerina[1], Eduardo Martínez[2]*

[1] Dpto. Electricidad y Electrónica, Facultad de Ciencias, University of Valladolid, E-47011 Valladolid, Spain.

[2] Dpto. Física Aplicada. University of Salamanca, Plaza de los Caídos s/n, E-38008, Salamanca, Spain.

* corresponding author: edumartinez@usal.es

S1. Symmetric and asymmetric double bit lines cases

In the main text we presented results considering that the FM is at the same vertical distance from the top and the bottom bit lines ($t_d^T = t_d^B = \frac{t_{FM}}{2} + 3$ nm, symmetric case). As this could be difficult to implement in real devices, here we show that that this is not essential for the suggested mechanism. To do it, we compare the perfect symmetric case ($t_d^T = t_d^B = \frac{t_{FM}}{2} + 3$ nm), where the top and bottom bit lines are placed at the same distance from the FM strip, to the case asymmetric case ($t_d^T \neq t_d^B$), where the top and bottom bit lines are also identical in shape and dimensions ($w_L = 200$ nm and $t_L = 50$ nm) but their centers are at different vertical distances from the FM layer (see suppl. Fig. S1). Along the x-axis, the center of the double bit line is at $x_L = -1536$ nm from the center of the FM layer. The center of top (bottom) line is at $z_L^{T(B)} = \pm\left(t_d^{T(B)} + \frac{t_L}{2}\right)$ above (below) the center of the FM layer ($z = 0$) for the symmetric case, where $t_d^T = t_d^B = \frac{t_{FM}}{2} + 3$ nm. Signs $+$ and $-$ correspond to the top (T) and bottom (B) bit lines respectively. Besides, for the asymmetric case, $t_d^T = \frac{t_{FM}}{2} + 3$ nm and $t_d^B = \frac{t_{FM}}{2} + 15$ nm. The resulting components of the Oersted field ($\mu_0 H_{Oe,x}$ and $\mu_0 H_{Oe,z}$) components are shown in the right graphs of Fig. S1(a) and (b) for the symmetric and asymmetric cases respectively. A small out-of-plane component ($\mu_0 H_{Oe,z}$ is observed for the asymmetric case. As in the case of the single bit line, $\mu_0 \left|H_{Oe,z}\right|$ reaches its

maximum close to the edges of the bit lines ($x = x_L \pm \frac{w_L}{2}$), and it decreases from the center of the bit lines (at $x = x_L$). However, $\mu_0 |H_{Oe,z}|$ rapidly decreases from the edge of the bit lines, as due to the *dipolar* behavior described in the text ($\mu_0 |H_{Oe,z}| \sim \frac{1}{|x-x_L|^3}$ for $|x - x_L| > \frac{w_L}{2}$).

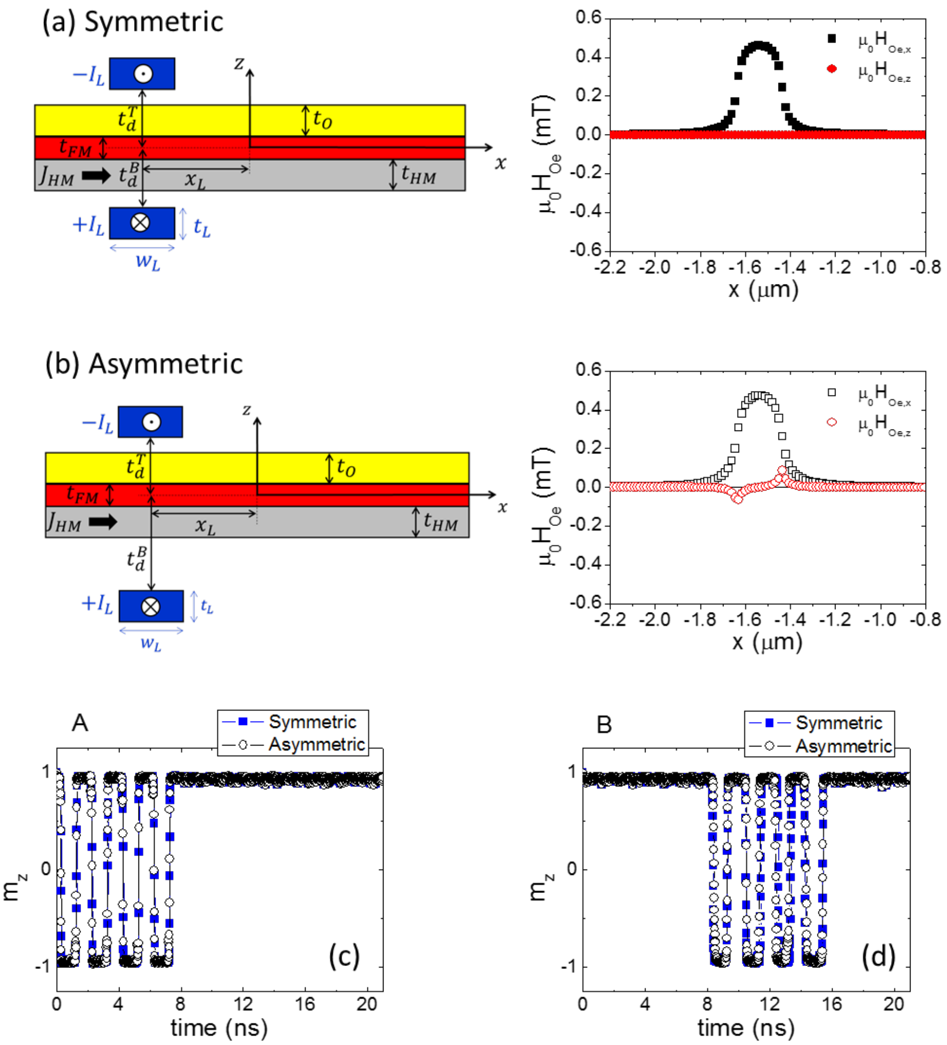

Figure S1. Influence of the FM distance to the top and the bottom bit lines. Left graphs in **(a)** and **(b)** represent the schematic symmetric and asymmetric configurations of the double bit line. The components of the Oersted field are plotted in right graphs. **(c)** and **(d)** correspond to the temporal evolution of the out-of-plane component of the magnetization at point A (c) and B (d) for the symmetric (full symbols) and asymmetric (open symbols) cases. All parameters and the bit sequence of current pulses are the same as in Fig. 5(a) of the main text.

In order to show that the nucleation proposed process is still valid for the asymmetric case, we have studied and compared the nucleation and shifting processes for the symmetric and asymmetric case by studying the same field and current pulses as in Fig. 5(a) in the main text, *i. e.*, to write and shift a sequence of 8 bit as “101010101”. The comparison of the temporal evolution of the out-of plane magnetization at points A and B clearly indicates that the dynamics, including both the DW nucleation and the displacement of nucleated DWs, is not sensitive to the asymmetric location of the bit lines (see Fig. S1 (c) and (d) for points A and B respectively).

S2. Single domain wall velocity

We have evaluated the dynamics of a single DW as a function of the J_{HM} for FM wires with two different widths $w = 96$ nm and $w = 192$ nm. Realistic conditions, where defects and imperfections are taken into account in the form of grains are assumed. The thickness and the material parameters are the same as described in the Methods section of the main text. The results of the DW velocity *vs* J_{HM} are shown in Fig. S2.

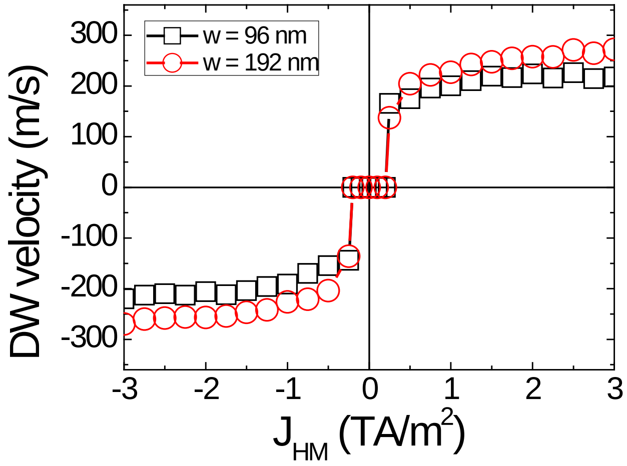

Figure S2. DW velocity as function of the injected current density through the HM.

S3. Movies of the dynamics processes of Fig. 5 in the main text

We provide two movies showing the dynamics of Fig. 5(a) and Fig. 5(b) in the main text.
